# Association between inflammatory burden index and prognosis in patients with coronary heart disease: A retrospective study

**DOI:** 10.1371/journal.pone.0325287

**Published:** 2025-07-07

**Authors:** Wen Lu, Xiaoqin Liao, Yan Jiang, Baolin Luo, Liangwan Chen, Yanjuan Lin

**Affiliations:** 1 School of Nursing, Fujian Medical University, Fuzhou, Fujian, China; 2 Department of Nursing, Fujian Medical University Union Hospital, Fuzhou, Fujian, China; 3 Department of Cardiovascular Surgery, Fujian Medical University Union Hospital, Fuzhou, Fujian, China; 4 Department of Neurology, Fujian Medical University Union Hospital, Fuzhou, Fujian, China; 5 Fujian Provincial Special Reserve Talents Laboratory, Fuzhou, Fujian, China; Azienda Ospedaliero Universitaria Careggi, ITALY

## Abstract

**Background:**

Increasing evidences indicate that systemic inflammation plays a significant role of adverse prognosis in patients with coronary heart disease (CHD). The inflammatory burden index (IBI) is a novel biomarker that reflects systemic inflammation. The aim of this study was to investigate the association between IBI and prognosis of CHD patients.

**Methods:**

In this retrospective analysis, data from 2453 CHD patients enrolled from December 2017 to December 2022.IBI was defined as neutrophil/lymphocyte*C-reactive protein, with patients categorized into four groups based on quartiles of baseline IBI levels. The primary outcome was adverse cardiovascular and cerebrovascular events (MACCEs) occurring during hospitalization, which included repeat revascularization, new-onset atrial fibrillation (NOAF), stroke, and all-cause in-hospital mortality. Multivariate logistic regression models and restricted cubic spline (RCS) analysis were used to investigate the association between IBI and prognosis of CHD patients.

**Results:**

High levels of IBI were associated with higher risk of MACCEs, especially when IBI ≥ 45.68, patients exhibited a higher risk of MACCEs (*P* < 0.05). After adjusting for baseline confounders, multivariate logistic regression analysis demonstrated that baseline IBI was an independent predictor for NOAF (Odds Ratio (OR): 2.05; 95% confidence interval (CI): 1.30–3.24; *P* = 0.002) and contrast-induced nephropathy (CIN) (OR: 1.95; 95%CI: 1.16–3.28; *P* = 0.012) in CHD patients, mainly driven by the highest quartile. In addition, RCS confirmed a linear relationship between IBI and NOAF (*P* for non-linear = 0.425) and a nonlinear relationship with CIN (*P* for non-linear = 0.032).

**Conclusion:**

IBI is a promising biomarker of systemic inflammation in CHD patients, where higher IBI levels are associated with adverse prognosis. These findings may aid clinicians in precise decision-making to improve outcomes in patients with CHD.

## Introduction

Coronary heart disease (CHD) is the most common cardiovascular disease worldwide, with a rising trend in both morbidity and mortality, and tend to be younger. It is reported that up to 9.1 million people die from CHD every year, accounting for 49.2% of all cardiovascular disease deaths and 16.3% of all-cause mortality, which brings a heavy burden to the patient’s family and society [1–3].Although interventional and pharmacologic therapies have substantially improved survival rates among CHD patients, the occurrence of adverse cardiovascular and cerebrovascular events (MACCEs) still has a significant negative impact on the prognosis and quality of life of patients [4–6]. Therefore, there is an urgent need for simple and effective biomarkers to facilitate early risk assessment and timely treatment.

The pathogenesis of CHD is complex and related to multiple factors, among which coronary atherosclerosis (AS) is its primary pathophysiological basis [7]. In recent years, evidence indicates that inflammatory responses and inflammatory factors play a pivotal role in promoting the formation, development and rupture of AS plaques [8]. Inflammatory factors promote the activation of endothelial cells, increase vascular permeability, and promote monocyte migration to the vascular wall, where they transform into macrophages and foam cells, ultimately contributing to plaque formation [9]. In addition, inflammation not only plays a contributory role in the development and progression of CHD, but also affects the process of repair and ventricular remodeling after myocardial ischemia in patients [10]. It is worth noting that the development of AS and the injury of endothelial function will promote inflammation in the body, forming a vicious cycle and affecting the prognosis of CHD patients [11]. Studies have shown that inflammation is closely related to the occurrence of MACCEs such as stroke and cardiovascular death, among patients with coronary artery disease(CAD), with systemic anti-inflammatory therapy shown to reduce the risk of MACCEs effectively [12–14]. Therefore, inflammatory biomarkers have significant clinical value in the diagnosis, treatment and prognosis of CHD [15–17].

Recently, Xie et al. [18] combined neutrophil to lymphocyte ratio (NLR) and C-reactive protein (CRP) to develop a novel systemic inflammatory marker named inflammatory burden index (IBI). Multiple studies have suggested that IBI is a reliable prognostic marker for different tumors and can effectively predict cachexia, mortality and complications of patients [19–21].Moreover, Song et al. [22] and Du et al. [23] demonstrated that the increase of IBI was strongly associated with an increased risk of adverse outcomes and the development of complications in patients with cerebrovascular diseases, and Du et al. found that IBI had the highest predictive accuracy and reclassification indexes. These evidences suggest that IBI is a potential predictor of poor prognosis in patients. However, studies specifically examining the relationship between IBI and cardiovascular disease are limited. Whether IBI has an effect on adverse prognosis in CHD patients remains unknown. Therefore, we conducted a retrospective study to explore the correlation between IBI and the prognosis of patients with CHD.

## Methods

### Study population

The study population consisted of patients diagnosed with CHD [24] at the Fujian Medical University Union Hospital from December 2017 to December 2022. The first time we accessed the data was on August 20, 2023. Exclusion criteria were as follows: (1) age < 18 years, (2) coronary artery bypass grafting (CABG) and other urgent surgeries, (3) severe hepatic and renal insufficiency, (4) patients suffering from systemic inflammatory diseases, malignant tumors, hematological diseases, autoimmune diseases, and severe infectious diseases, and (5) patients with incomplete medical records. This study adhered to the principles of the Declaration of Helsinki and received approval from the Ethics Committee of Fujian Medical University Union Hospital (Ethics approval No. 2023KY032). Obtaining informed consent was waived for this study because it was a retrospective study.

### Data collection

Data were collected through the hospital electronic medical record system, including general clinical data, laboratory tests, medicine care, and clinical outcomes. General clinical data included age, gender, smoking, drinking, systolic blood pressure (SBP), diastolic blood pressure (DBP), body mass index (BMI), left ventricular ejection fraction (LVEF), heart rate (HR), hypertension, diabetes mellitus (DM), Hyperlipidemia, chronic kidney disease (CKD), prior myocardial infarction (MI), prior percutaneous coronary intervention (PCI), prior CABG, types of CHD. Laboratory tests collected white blood cell count, red blood cell count, neutrophil, lymphocyte, platelet count, hemoglobin count, fasting blood glucose (FBG), total cholesterol (TC), triglycerides (TG), low-density lipoprotein cholesterol (LDL-L), high-density lipoprotein cholesterol (HDL-L), fibrinogen, serum creatinine (Scr), and blood urea nitrogen (BUN). Medical treatment included PCI and pharmacologic therapy, including statins, dual antiplatelet therapy dual (DAPT), diuretics, β-blockers, angiotensin-converting enzyme inhibitors/angiotensin receptor blockers/angiotensin receptor enkephalinase inhibitors (ACEI/ARB/ARNI). Fasting venous blood was collected for the first time in the morning after admission to all patients, and the blood index level was determined.

### Calculation of IBI

IBI was defined as (Neutrophil count/Lymphocyte count) *C-reactive protein [18].

### Outcome measures

The primary outcome was MACCEs occurring during hospitalization, which included repeat revascularization, new-onset atrial fibrillation (NOAF), stroke, and all-cause in-hospital mortality. All-cause hospital mortality was defined as death from any cause during hospitalization. Repeat revascularization was defined as a second stent placement in addition to the first stent during hospitalization and excluded patients who underwent CABG procedures. New-onset atrial fibrillation was defined as no history of atrial fibrillation, which was documented using routine electrocardiograms, ambulatory electrocardiograms, and electrocardiographic monitoring equipment during the outpatient or hospitalization period. Secondary outcomes were contrast-induced nephropathy (CIN) and 1-year all-cause readmission rate. CIN was defined as an increase in serum creatinine concentration of 25% or 44.2 μmol/L from baseline within 48–72 hours of exposure to a contrast agent, with the exclusion of other factors that could have contributed to acute kidney injury. The outcomes were collected primarily through an electronic medical record system. Patients included in this study were followed up for 1 year.

### Statistical analysis

Statistical analyses were performed using SPSS 26.0 (IBM Corp., Armonk, NY, USA) and R 4.4.1 software (R Statistical Calculation Foundation, Vienna, Austria), and a two-sided *P* < 0.05 was considered to be statistically significant differences.

Categorical variables were expressed as frequencies and percentage, and were analyzed using the chi-square test. Continuous variables were assessed for normal distribution, and variables conforming to normal distribution were expressed as the mean with standard deviation (SD), and were analyzed using the independent samples t-test. While non-normally distributed variables were expressed as median (IQR), and were analyzed using the Mann-Whitney U-test. The analysis proceeded in three stages. First, the inflammatory biomarker index (IBI) was divided into quartiles for subgroup comparison. Next, potential risk factors for clinical outcomes were identified through univariate logistic regression. Finally, independent predictors of clinical outcomes were determined using multivariate logistic regression models. Multivariate Cox regression models were used to assess all-cause readmission rate. Restricted cubic spline (RCS) analyses were used to explore the dose-response relationship between IBI and clinical outcomes. The optimal model was selected by Akaike information criterion (AIC). Variables with more than 20% missing values were not included in the analysis. Multiple interpolation was used to supplement variables with less than 20% missing values.

## Results

### Patient characteristics

**Fig 1:**
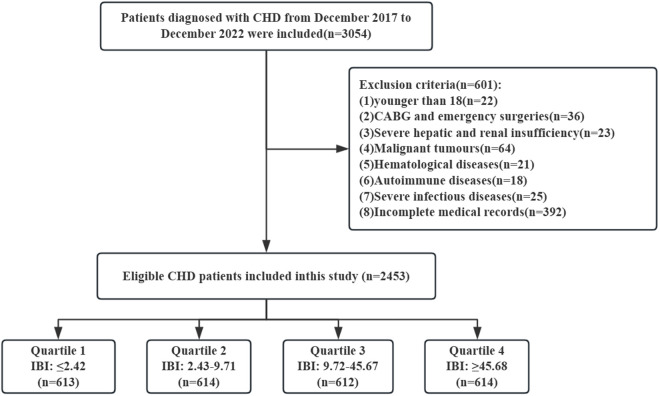
Flow chart of patient selection. Abbreviations: CHD, coronary heart disease; CABG, coronary artery bypass surgery; IBI, inflammatory burden index.

A total of 2453 patients were finally enrolled in this study [1], and the flow chart of patient inclusion is shown in Fig 1. The mean age of the 2453 patients was 64.49 ± 10.59, and 78.4% were male. Patients were divided into four groups according to the IBI quartile for baseline characteristics analysis (Table 1). As the IBI quartiles increased, the incidence of CKD in CHD patients increased, but was accompanied by lower SBP and LVEF, and a decreased history of MI and PCI. Interestingly, the incidence of hyperlipidemia peaked in the third quartile.

**Table 1:** Characteristics of CHD patients.

Variables	IBI				P Value
	Quartile 1 (≤2.42)	Quartile 2 (2.43-9.71)	Quartile 3 (9.72-45.67)	Quartile 4 (≥45.68)	
	(n = 613)	(n = 614)	(n = 612)	(n = 614)	
**Demographic and clinical characteristics**					
Age, years, M (SD)	63.67 ± 9.91	63.67 ± 9.91	65.01 ± 10.51	64.86 ± 10.86	0.115
Male gender, n (%)	479 (78.1)	477 (77.7)	484 (79.1)	483 (78.7)	0.940
Current smoking, n (%)	215 (35.1)	213 (34.7)	229 (37.4)	239 (38.9)	0.616
Current drinking, n (%)	42 (6.9)	48 (7.8)	48 (7.8)	54 (8.8)	0.910
SBP, mmHg, M (SD)	132.02 ± 18.58	131.14 ± 19.60	130.07 ± 19.34	128.92 ± 21.46	0.041
DBP, mmHg, M (SD)	79.52 ± 12.05	78.97 ± 12.54	78.58 ± 30.70	78.24 ± 13.61	0.668
BMI, kg/m2, M (SD)	24.12 ± 3.54	24.65 ± 2.92	24.42 ± 2.71	24.01 ± 2.69	<0.001
LVEF, M (SD)	62.79 ± 9.82	60.60 ± 10.91	58.57 ± 12.22	55.94 ± 12.86	<0.001
HR, M (SD)	74.76 ± 26.35	74.57 ± 11.91	76.29 ± 14.22	80.72 ± 17.07	<0.001
Hypertension, n (%)	383 (62.5)	413 (67.3)	390 (63.7)	387 (63.0)	0.294
DM, n (%)	237 (38.7)	233 (37.9)	249 (40.7)	224 (36.5)	0.498
Hyperlipemia, n (%)	203 (33.1)	227 (37.0)	243 (39.7)	170 (27.7)	<0.001
CKD, n (%)	31 (5.1)	47 (7.7)	67 (10.9)	104 (16.9)	<0.001
Prior MI, n (%)	86 (14.0)	52 (8.5)	43 (7.0)	28 (4.6)	<0.001
Prior PCI, n (%)	172 (28.1)	141 (23.0)	116 (19.0)	78 (12.7)	<0.001
Prior CABG, n (%)	17 (2.8)	16 (2.6)	10 (1.6)	15 (2.4)	0.566
Types of CHD, n (%)					0.012
Acute coronary syndrome	444 (72.4)	444 (72.3)	462 (75.5)	412 (67.1)	
Stable coronary heart disease	169 (27.6)	170 (27.7)	150 (24.5)	202 (32.9)	
**Baseline chemistry**					
WBC, 10^9/L, M (SD)	6.42 ± 1.73	7.25 ± 2.38	7.97 ± 2.74	9.32 ± 3.74	<0.001
RBC, 10^12/L, M (SD)	4.52 ± 0.69	4.45 ± 0.64	4.40 ± 0.67	4.24 ± 0.71	<0.001
Lymphocyte, 10^9/L, M (SD)	3.76 ± 1.34	4.61 ± 1.90	5.44 ± 2.49	6.89 ± 3.66	<0.001
NE, 10^9/L, M (SD)	2.00 ± 0.76	1.92 ± 1.03	1.78 ± 0.62	1.62 ± 0.70	<0.001
Platelet, 10^9/L, M (SD)	217.91 ± 59.70	227.30 ± 62.61	235.81 ± 76.47	238.67 ± 82.40	<0.001
Hemoglobin, g/L, M (SD)	136.70 ± 16.39	134.92 ± 18.14	132.16 ± 18.67	127.25 ± 21.69	<0.001
FBG, mmol/L, M (SD)	5.90 ± 2.01	6.29 ± 2.87	6.56 ± 2.92	6.81 ± 3.25	<0.001
TC, mmol/L, M (SD)	4.09 ± 1.21	4.31 ± 1.49	4.33 ± 1.18	4.39 ± 1.25	0.023
TG, mmol/L, MED (IQR)	0.99 (1.34–1.86)	1.09 (1.56–2.13)	1.07 (1.44–2.00)	1.01 (1.40–1.90)	<0.001
HDL-C, mmol/L, M (SD)	1.08 ± 0.27	1.02 ± 0.31	1.01 ± 0.26	1.01 ± 0.28	<0.001
LDL-C, mmol/L, M (SD)	2.59 ± 1.10	2.77 ± 1.18	2.83 ± 1.08	2.90 ± 1.08	<0.001
Fibrinogen, g/L, M (SD)	3.26 ± 0.70	3.69 ± 1.00	3.99 ± 1.08	4.55 ± 1.58	<0.001
Scr, mg/dl, M (SD)	77.55 ± 21.71	85.72 ± 33.67	95.21 ± 73.23	107.09 ± 89.80	<0.001
BUN, mg/dl, M (SD)	5.60 ± 1.90	6.11 ± 2.73	6.41 ± 3.86	6.97 ± 4.15	<0.001
**Medicine care**					
Diuretics, n (%)	82 (13.4)	101 (16.4)	146 (23.9)	220 (35.8)	<0.001
DAPT, n (%)	564 (92.0)	521 (84.9)	474 (77.5)	391 (63.7)	<0.001
β-blockers, n (%)	361 (58.9)	347 (56.5)	332 (54.2)	303 (49.3)	0.006
Statins, n (%)	552 (90.0)	511 (83.2)	481 (78.6)	398 (64.8)	<0.001
ACEI/ARB/ARNI, n (%)	246 (40.1)	227 (37.0)	191 (31.2)	163 (26.5)	<0.001

Abbreviations: SBP, systolic blood pressure; DBP, diastolic blood pressure; BMI, body mass index; LVEF, left ventricular ejection fraction; HR, heart rate; DM, diabetes mellitus; CKD, chronic kidney disease; MI, myocardial infarction; PCI, percutaneous coronary intervention; CABG, coronary artery bypass graft surgery; CHD, coronary heart disease; WBC, White blood cells; RBC, red blood cells; NE, neutrophiles; FBG, fasting blood glucose; TC, total cholesterol; TG, triglyceride; HDL-C, high-density lipoprotein cholesterol; LDL-C, low-density lipoprotein cholesterol; Scr, serum creatinine; BUN, blood urea nitrogen; DAPT, dual antiplatelet therapy; ACEI or ARB or ARNI, angiotensin-converting enzyme inhibitors or angiotensin receptor blockers or angiotensin receptor neprilysin inhibitors.

Compared to the first three groups, patients with the highest IBI level had the highest levels of white blood cells, lymphocytes, platelets, FBG, TC, LDL-L, fibrinogen, Scr and BUN, but the highest levels of TG in second quartile. In addition, we observed that the levels of red blood cells, neutrophils, hemoglobin, and HDL-L decreased as the IBI quartile increased.

In terms of medicine care, patients in the highest quartile were treated with DAPT more frequently than patients in the lowest quartile array. However, diuretics, statins, β-blockers, and ACEI/ARB/ARNI were used most frequently in the lowest quartile.

### Association between IBI and CHD patient outcomes

In hospital outcomes for CHD patients, the risk of MACCEs increased with the increase of IBI quartile, including the incidence of NOAF (*P* = 0.003) and all-cause in-hospital mortality (*P* < 0.001) was statistically significant. However, no difference was observed between repeated revascularization (*P* = 0.814) and stroke (*P* = 0.922) in the four groups.

In addition, we observed a significant increase in CIN risk in the highest quartile (14.7%, *P* < 0.001), but there was no difference in all-cause readmission rates among the four groups (*P* = 0.105) (Table 2).

**Table 2:** Clinical Outcomes of CHD Patients stratified by IBI quartiles.

Outcomes	IBI				*P* Value
	Quartile 1 (≤2.42)	Quartile 2 (2.43-9.71)	Quartile 3 (9.72-45.67)	Quartile 4 (≥45.68)	
	(n = 613)	(n = 614)	(n = 612)	(n = 614)	
**MACCEs**					
Repeated revascularization	35 (5.7)	38 (6.2)	43 (7.0)	40 (6.5)	0.814
NOAF	39 (6.4)	57 (9.3)	58 (9.5)	77 (12.5)	0.003
Stroke	38 (6.2)	38 (6.2)	35 (5.7)	41 (6.7)	0.922
All-cause in-hospital mortality	1 (0.2)	3 (0.5)	9 (1.5)	20 (3.3)	<0.001
**CIN**	32 (5.2)	24 (3.9)	40 (6.5)	90 (14.7)	<0.001
**Readmission**	193 (31.5)	199 (32.4)	185 (24.9)	162 (30.1)	0.105

Abbreviations: IBI, inflammatory burden index; MACCEs, major adverse cardiac and cerebrovascular events; NOAF, new-onset atrial fibrillation; CIN, contrast-Induced nephropathy.

Multivariate logistic regression models were used to further explore the association between IBI and outcome indicators (Table 3). Model I was an unadjusted model, and as IBI increased, so did the risk of MACCEs (mainly NOAF and all-cause in-hospital mortality). Model II adjusted for sex and age, and the results were consistent with Model I. Model III adjusted for statistically significant differences in the baseline analysis (*P* < 0.05) and included sex, age, SBP, BMI, hyperlipidemia, LVEF, HR, CKD, prior MI, prior PCI, types of CHD, red blood cells, lymphocytes, neutrophils, platelets, hemoglobin, FBG, TC, LDL-L, HDL-L, fibrinogen, Scr, BUN, diuretics, DAPT, β-blockers, statins, ACEI/ARB/ARNI. It is worth noting that leukocytes and total cholesterol were not included in the analysis due to covariance (variance in stimulation factor (VIF)>5). The results further confirmed that increased IBI levels were independently associated with increased MACCEs (*P* < 0.05). Specifically, high IBI was an independent risk factor for NOAF in CHD patients (OR: 2.05; 95%CI: 1.30–3.24; *P* = 0.002). Although we observed that IBI levels were significantly associated with all-cause hospital mortality in a previous analysis (*P* < 0.001), this result was not further confirmed after adjusting for baseline confounders (OR: 5.37; 95%CI: 0.53–54.90; *P* = 0.156). However, no difference (*P* > 0.05) was observed between repeat revascularization (*P* = 0.739) and stroke (*P* = 0.936) in the different IBI groups.

**Table 3:** Risk of Clinical Outcomes according to IBI quartiles.

IBI	Model Ⅰ		Model Ⅱ		Model Ⅲ	
	OR/HR (95% CI)	*P*	OR/HR (95% CI)	*P*	OR/HR (95% CI)	*P*
**MACCEs**						
Repeated revascularization						
Quartile1	Reference		Reference		Reference	
Quartile2	1.09 (0.68–1.75)	0.723	1.10 (0.68–1.76)	0.708	0.89 (0.54–1.46)	0.631
Quartile3	1.25 (0.79–1.98)	0.346	1.25 (0.79–1.99)	0.339	0.85 (0.51–1.42)	0.525
Quartile4	1.15 (0.72–1.84)	0.556	1.15 (0.72–1.84)	0.550	0.70 (0.38–1.31)	0.265
*P* for trend		0.815		0.810		0.739
NOAF						
Quartile1	Reference		Reference		Reference	
Quartile2	1.51 (0.99–2.30)	0.058	1.37 (0.89–2.10)	0.157	1.47 (0.94–2.30)	0.088
Quartile3	1.54 (1.01–2.35)	0.045	1.40 (0.91–2.16)	0.122	1.54 (0.97–2.43)	0.067
Quartile4	2.11 (1.41–3.16)	<0.001	1.99 (1.32–3.00)	0.001	2.05 (1.30–3.24)	0.002
*P* for trend		0.004		0.010		0.024
Stroke						
Quartile1	Reference		Reference		Reference	
Quartile2	1.00 (0.63–1.59)	0.994	0.94 (0.59–1.50)	0.789	0.94 (0.58–1.53)	0.806
Quartile3	0.92 (0.57–1.47)	0.723	0.86 (0.54–1.39)	0.544	0.85 (0.50–1.42)	0.523
Quartile4	1.08 (0.69–1.71)	0.733	1.03 (0.65–1.64)	0.891	0.90 (0.51–1.60)	0.729
*P* for trend		0.922		0.885		0.936
All-cause in-hospital mortality						
Quartile1	Reference		Reference		Reference	
Quartile2	3.01 (0.31–28.97)	0.341	3.02 (0.31–29.07)	0.340	1.62 (0.14–19.21)	0.704
Quartile3	9.13 (1.15–72.32)	0.036	9.19 (1.16–72.75)	0.036	4.61 (0.46–46.02)	0.193
Quartile4	20.61 (2.76–154.03)	0.003	20.68 (2.77–154.58)	0.003	5.37 (0.53–54.90)	0.156
*P* for trend		<0.001		<0.001		0.237
**CIN**						
Quartile1	Reference		Reference		Reference	
Quartile2	0.74 (0.43–1.27)	<0.273	0.72 (0.42–1.24)	0.239	0.68 (0.38–1.21)	0.187
Quartile3	1.27 (0.79–2.06)	0.329	1.26 (0.78–2.03)	0.355	1.02 (0.60–1.74)	0.953
Quartile4	3.12 (2.05–4.75)	<0.001	3.12 (2.05–4.76)	<0.001	1.95 (1.16–3.28)	0.012
*P* for trend		<0.001		<0.001		<0.001
**Readmission**						
Quartile1	Reference		Reference		Reference	
Quartile2	0.98 (0.80–1.20)	0.843	0.97 (0.79–1.19)	0.764	1.06 (0.83–1.37)	0.630
Quartile3	0.96 (0.78–1.18)	0.694	0.95 (0.77–1.16)	0.617	0.94 (0.72–1.22)	0.629
Quartile4	1.18 (0.95–1.45)	0.129	1.18 (0.96–1.46)	0.117	0.79 (0.58–1.08)	0.142
*P* for trend		0.230		0.174		0.242

Model I: unadjusted.

Model Ⅱ: adjusted for age and male sex.

Model Ⅲ: adjusted for Model Ⅱ plus other risk factors.

Abbreviations: OR, odds ratio; HR, hazard ratio; CI, confidence interval; IBI, inflammatory burden index; MACCEs, major adverse cardiac and cerebrovascular events; NOAF, new-onset atrial fibrillation; CIN, contrast-Induced nephropathy.

Furthermore, in this study, IBI levels were significantly associated with the risk of CIN after adjusting for baseline confounders (OR: 1.95; 95% CI: 1.16–3.28; *P* = 0.012). The OR were 0.68, 1.02 and 1.95 for the second quartile, the third quartile and the fourth quartile compared to the first quartile. There was no statistically significant difference in all-cause readmission rates among IBI groups (*P* = 0.237) (Table 3).

**Fig 2:**
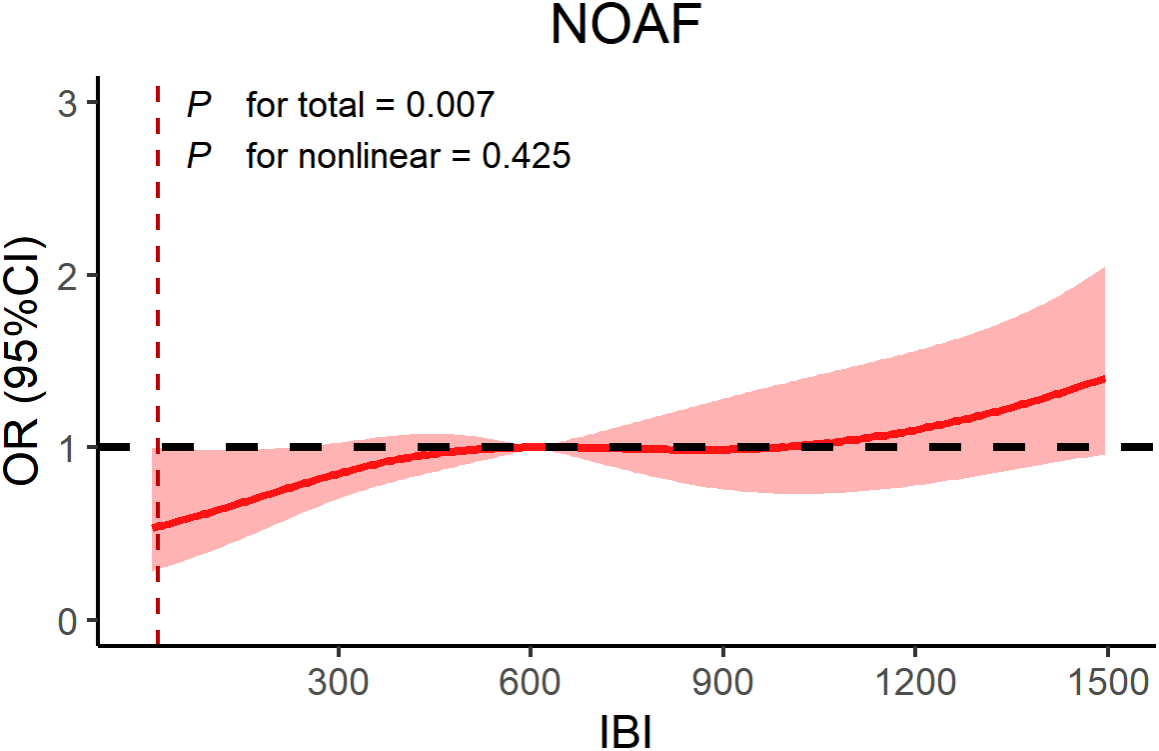
RCS model showing the associations of IBI with NOAF. Abbreviations: NOAF, new-onset atrial fibrillation; OR, odds ratio; CI, confidence interval; IBI, inflammatory burden index.

**Fig 3:**
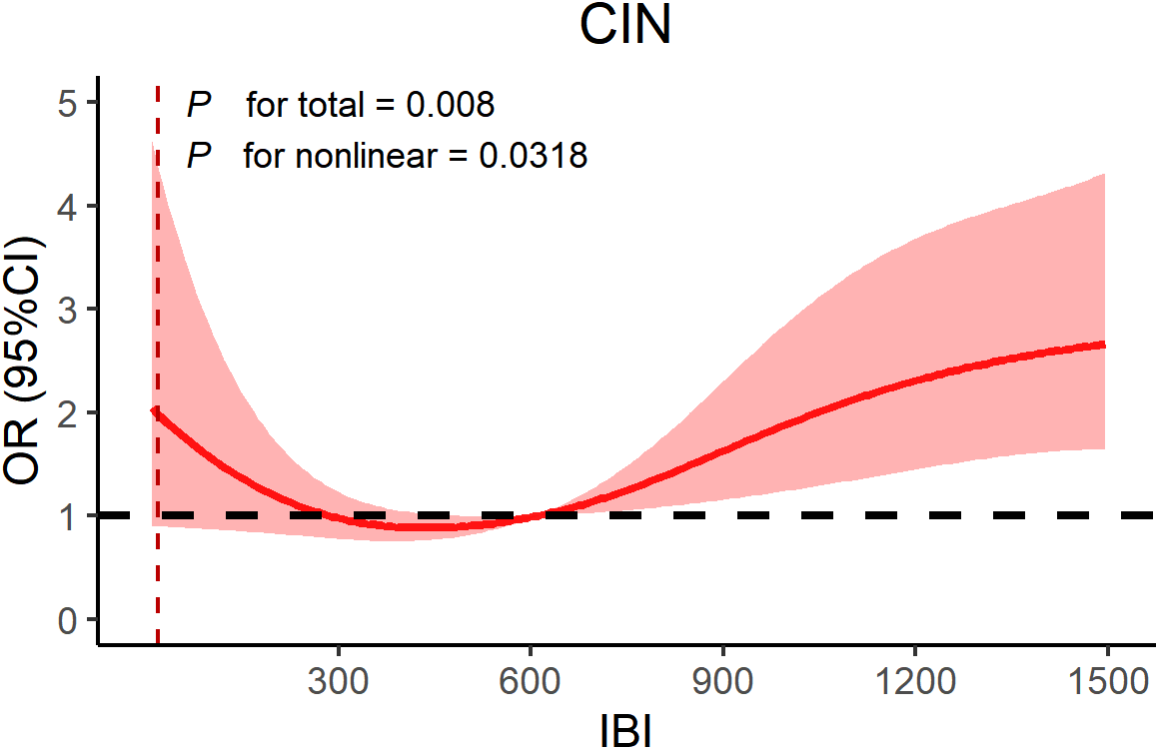
RCS model showing the associations of IBI with CIN. Abbreviations: CIN, contrast-Induced nephropathy; OR, odds ratio; CI, confidence interval; IBI, inflammatory burden index.

RCS analysis was used to assessed the association between IBI and NOAF, IBI and CIN. We observed a linear relationship between continuous IBI and NOAF (*P* for linear = 0.425) (Fig 2), but a nonlinear relationship between continuous IBI and CIN risk (*P* for linear = 0.0318) (Fig 3). The risk of CIN increased with increasing levels of IBI.

## Discussion

To the best of our knowledge, this is the first study to investigate IBI and prognosis in patients with CHD. The results of the study showed that higher levels of IBI were associated with an increased risk of NOAF and CIN compared to lower levels of IBI, which were further confirmed after adjusting for potential confounders. In addition, we investigated the dose-response relationship between IBI and NOAF and CIN in CHD patients, and the RCS confirmed a linear relationship between IBI and NOAF and a nonlinear relationship with CIN. Therefore, IBI may be a reliable independent risk factor for assessing prognosis in CHD patients.

Atherosclerosis (AS) is the primary pathophysiological basis of CHD, and the stability of AS plaques is crucial in the occurrence and development of CHD [11]. In recent decades, research data have shown that the balance between pro-inflammatory and anti-inflammatory responses in the immune system plays an important role in plaque stability and disease progression [7,25]. There is a complex pathophysiological relationship between AS, inflammation and CHD, with inflammatory responses and inflammatory factors are present throughout the whole process of the occurrence and development of CHD [26]. Therefore, inflammatory markers appear to be the most promising as a strong predictor of prognosis in patients with CHD. As a novel marker of systemic inflammation, IBI has been confirmed to be closely related to patient prognosis [22]. However, the mechanism related to the prognosis of patients with IBI and CHD remains unclear. Previous studies have shown that neutrophils secrete a large number of inflammatory mediators, anaerobic free radicals and chemotactic agents, which are focused on the site of plaque erosion, leading to endothelial cell damage and activation of macrophages, which initiates AS and promotes plaque progression [27]. The process of AS leads to apoptosis of lymphocytes co-existing in plaques, which aggravate plaque load and increases the risk of poor prognosis [28]. Neutrophils and lymphocytes combine information on innate and adaptive immunity and are effective biomarkers of prognosis in CHD patients, providing new insights into inflammatory AS [29–31]. In addition, CRP not only serves as a biomarker for AS but also contributes to thrombosis risk by impairing endothelial function, promoting endothelial dysfunction, and inducing the release of circulating endothelial cells and endothelial microparticles [32,33]. A large number of studies have demonstrated that abnormal neutrophils, lymphocytes and CRP are associated with an increased risk of poor prognosis in patients with CAD [34–37]. IBI combines the advantages of different inflammatory markers, and abnormal IBI may indicate more severe endothelial cell damage and plaque load. Therefore, IBI may have potential prognostic value for the prognosis of CHD patients.

Previous studies have shown that the level of systemic inflammation is strongly associated with MACCEs in CHD patients [38]. Consistently, in our study, we found that higher levels of IBI were associated with a higher incidence of MACCEs in CHD patients. An observational study noted that systemic inflammatory biomarkers were independent predictors of MACCEs in CHD patients undergoing non-cardiac surgery [36]. Wada et al. observed that a high NLR was associated with poor prognosis in patients with CAD [39]. A cohort study suggested that with the use of high-sensitivity C-reactive protein (hs-CRP) alone, the risk of poor prognosis in patients may be incorrectly assessed, and that the risk of MACCEs was increased only when NLR was increased in concert with hs-CRP [40]. IBI was proposed by Xie et al. to reflect systemic inflammatory status and is a promising prognostic biomarker [18]. Recently, more and more studies have focused on the correlation between IBI and prognosis in different populations [22,23,41]. Our findings also support this conclusion, with elevated IBI reflecting an enhanced inflammatory response in patients with CHD. All these evidences suggest that IBI is a reliable inflammatory biomarker for predicting the prognosis of CHD patients.

Our study found a positive correlation between baseline IBI and NOAF in CHD patients, driven by the highest quartile of IBI, and a linear and increasing relationship between the IBI and NOAF. Similar to our findings, Nortamo et al. found that hs-CRP was closely associated to the occurrence of NOAF in CAD [42]. Ali et al. confirmed that the systemic immune inflammatory index is a predictor of NOAF after ST-segment elevation MI [43]. Inflammatory markers that are prevalent in CHD patients, such as leukocytes and CRP, are thought to be closely associated with electrophysiologic stability of the heart [36,37,44]. Inflammatory mediators increase the risk of atrial fibrillation (AF) by remodeling cardiac tissue and neurophysiology, leading to structural changes in the cardiac muscle and the conductive system, altering the electrophysiological properties of the atria [45,46]. Notably, AF also induces AS and promotes thrombosis, further promoting or exacerbating CHD [47]. Therefore, it can be hypothesized that controlling the inflammatory response may help reduce the risk of NOAF in CHD patients. In addition, our study showed that IBI was associated with all-cause in-hospital mortality, but there was no significant correlation between IBI and all-cause in-hospital mortality after adjusting for baseline confounders. This contradicts the results of existing studies [23] which may be caused by differences in inflammatory response, duration of inflammation and follow-up time of outcomes in different disease populations. Therefore, long-term large-sample studies are needed for further exploration in the future.

In recent years, with the extensive development of interventional surgery in clinical practice, the incidence of CIN caused by the sharp increase in the use of contrast agents also shows a corresponding increasing trend, which seriously affects the prognosis of patients [48]. Factors related to CIN include renal medullary ischemia and hypoxia, oxidative stress, endothelial dysfunction and inflammatory response, etc. however, the formation mechanism of CIN is complex and still completely unclear [49]. Currently, most studies agree that inflammatory response plays a crucial role in the onset and progression of CIN [48–50]. Some inflammatory markers, such as NLR, CRP and red blood cell distribution width, have been proposed to be closely related to the occurrence of CIN after PCI [51]. Butt et al. Found that NLR had good accuracy in predicting CIN in CAD [52]. Consistently, after adjusting for confounders, we observed that high levels of IBI were an independent risk factor for CIN in CHD patients, and RCS results further revealed a nonlinear relationship between IBI and CIN risk. This provides further support for the predictive value of inflammatory markers in CIN. Previous animal experiments have found inflammatory factors increase significantly after contrast agent administration, causing kidney tissue damage and thus inducing acute kidney injury. In addition, nephroenzymes and antithrombin can improve kidney injury by inhibiting inflammatory response [53,54]. We also noticed that low IBI was accompanied by high CIN, which may be associated with lymphocytosis and can exacerbate renal injury by releasing pro-inflammatory cytokines, activating NLRP3 inflammatory vesicles, cytotoxicity, and immune cell infiltration further exacerbating inflammation [55,56]. Therefore, the systemic inflammation reflected by IBI may, to some extent, reflect the kidney injury and help prevent CIN. Therefore, early identification of high-risk patients can help provide clinical potential intervention strategies to reduce the incidence of CIN and improve patient prognosis.

### Limitations

In this study, we not only evaluated the prognostic value of IBI in CHD patients, but also explored the dose-response relationship between IBI and prognosis to provide strategies for future prevention and improvement of CHD prognosis. However, our study has several limitations. First, this study was a retrospective, single-center study. There may be deficiencies in patient selection, study design and generalization. Secondly, the level of IBI changes dynamically, and the correlation between the dynamic changes of IBI and prognosis was not explored in this study. Therefore, future multi-center, prospective studies are needed for validation.

## Conclusion

We applied the inflammatory biomarker index (IBI) to patients with coronary heart disease (CHD) for the first time and confirmed that it is a valid marker for predicting prognosis in this population. IBI was independently associated with the risk of NOAF and CIN in CHD patients. Additionally, a nonlinear relationship was observed between IBI and CIN risk in these patients. Therefore, IBI may be a novel inflammatory marker to predict the prognosis of CHD patients.
